# Ovary Signals for Pollen Tube Guidance in Chalazogamous *Mangifera indica* L.

**DOI:** 10.3389/fpls.2020.601706

**Published:** 2021-02-10

**Authors:** Jorge Lora, Veronica Perez, Maria Herrero, Jose I. Hormaza

**Affiliations:** ^1^Subtropical Fruit Crops Department, Instituto de Hortofruticultura Subtropical y Mediterránea La Mayora (IHSM La Mayora-CSIC-UMA), Algarrobo-Costa, Spain; ^2^Unidad Técnica del Instituto de Productos Naturales y Agrobiología, IPNA-CSIC, Laboratorio de Agrobiología Juan José Bravo Rodríguez (Cabildo de La Palma), Santa Cruz de La Palma, Spain; ^3^Instituto de Productos Naturales y Agrobiologia (IPNA-CSIC), San Cristóbal de La Laguna, Spain; ^4^Pomology Department, Estación Experimental Aula Dei-CSIC, Zaragoza, Spain

**Keywords:** *Mangifera indica*, chalazogamy, pollen tube guidance, GABA, γ-aminobutyric acid, ovary signals, ponticulus

## Abstract

Most flowering plants show porogamy in which the pollen tubes reach the egg apparatus through the micropyle. However, several species show chalazogamy, an unusual pollen tube growth, in which the pollen tubes reach the embryo sac through the chalaza. While ovary signals for pollen tube growth and guidance have been extensively studied in porogamous species, few studies have addressed the process in chalazogamous species such as mango (*Mangifera indica* L.), one of the five most important fruit crops worldwide in terms of production. In this study, we characterize pollen–pistil interaction in mango, paying special attention to three key players known to be involved in the directional pollen tube growth of porogamous species such as starch, arabinogalactan proteins (AGPs), and γ-aminobutyric acid (GABA). Starch grains were observed in the style and in the ponticulus at anthesis, but their number decreased 1 day after anthesis. AGPs, revealed by JIM8 and JIM13 antibodies, were homogenously observed in the style and ovary, but were more conspicuous in the nucellus around the egg apparatus. GABA, revealed by anti-GABA antibodies, was specifically observed in the transmitting tissue, including the ponticulus. Moreover, GABA was shown to stimulate *in vitro* mango pollen tube elongation. The results support the heterotrophic growth of mango pollen tubes in the style at the expense of starch, similarly to the observations in porogamous species. However, unlike porogamous species, the micropyle of mango does not show high levels of GABA and starch, although they were observed in the ponticulus and could play a role in supporting the unusual pollen tube growth in chalazogamous species.

## Introduction

In flowering plants, after pollen germination, the pollen tubes face a long journey before the two male sperms meet the female gametophyte or embryo sac, which at maturity usually contains seven cells (two synergids, the egg cell, the central cell, and three antipodal cells), embedded in the sporophytic tissues of the ovule. Pollen germination and pollen tube growth take place within the carpel. Pollen grains first land on the stigma where they germinate and pollen tube growth takes place along the style, until reaching the ovarian cavity, the locule, at the base of the style. At this point, most flowering plants show porogamy in which pollen tubes reach the egg apparatus through the micropyle in order to achieve the double fertilization process typical of angiosperms (Maheshwari, 1950; Lora et al., 2016). The micropyle is generally formed by two integuments (bitegmic ovules), although some species show a single integument (unitegmic ovules) (Lora et al., 2015).

Pollen tube growth is a high energy–demanding process (Selinski and Scheibe, 2014), which is dependent on the sporophytic tissues of the pistil. The style generally shows a secretion that provides nutritive support for pollen tube growth (Herrero and Dickinson, 1979; Herrero and Hormaza, 1996; Lora et al., 2016; Gotelli et al., 2017). Several molecules have been identified as major contributors to pollen tube growth and guidance in the stigma and style. Those include lipids (Wolters-Arts et al., 1998; Hiscock, 2008), reactive oxygen species (McInnis et al., 2006; Hiscock, 2008; Zafra et al., 2010), esterases (Hiscock et al., 2002), or Ca^2+^ gradients (Ge et al., 2009). Stylar secretion is also rich in polysaccharides that have been reported in numerous porogamous angiosperms (Gotelli et al., 2017). Indeed, starch grains are generally observed in the cells of the transmitting tissue (Herrero and Hormaza, 1996; Lora et al., 2016). A common observation in several angiosperms is that starch grains disappear as the pollen tubes grow through the styles (Herrero and Dickinson, 1979; Cruden and Lyon, 1985; Herrero and Arbeloa, 1989; Gonzalez et al., 1996; Martinez-Palle and Herrero, 1998; Stephenson et al., 2003; Hu et al., 2019), supporting the nutritive role of carbohydrates for pollen tube growth. Thus, the presence of starch grains defines the pathway for pollen tube growth, and this has been barely studied in chalazogamous species.

Arabinogalactan proteins (AGPs) have been observed along the pollen tube pathway in a number of unrelated porogamous species. AGPs are extensively glycosylated hydroxyproline (Hyp)–rich proteins (Silva et al., 2020) that have been associated with the acquisition of stigmatic receptivity (Losada and Herrero, 2012; Losada et al., 2014) and pollen tube growth in the style (Cheung et al., 1995; Wu et al., 1995; Losada and Herrero, 2014). Moreover, in the ovary, where an intense pollen–pistil interaction occurs (Herrero, 2000, 2001), AGPs also appear to mediate access of the pollen tubes to the ovules at the obturator (Losada and Herrero, 2017) and support pollen tube penetration to the ovule through the micropyle (Coimbra et al., 2007; Lora et al., 2019; Losada and Herrero, 2019). AGPs have also been observed around the embryo sac and the egg apparatus in species with crassinucellate ovules (Coimbra and Salema, 1997; Lora et al., 2019; Losada and Herrero, 2019), in which the embryo sac is buried in two or more cell layers of the nucellus.

γ-aminobutyric acid (GABA, a ubiquitous glutamate derivative) has also been reported in the ovule, specifically in the tip of the inner integument that forms the micropyle (Palanivelu et al., 2003; Lora et al., 2019). Its presence has been related to prevention of the entrance of multiple pollen tubes in the ovule (Palanivelu et al., 2003; Lora et al., 2019) and to the modulation of pollen tube growth, modifying Ca^2+^-permeable channels of the pollen tube cell walls (Yu et al., 2014). Several molecules that are key for pollen tube attraction have been reported in the embryo sac [reviewed in Higashiyama and Takeuchi (2015), Higashiyama and Yang (2017) and Johnson et al. (2019)]. Most of these studies have been performed in the model plant *Arabidopsis thaliana* and in *Torenia fournieri* that show a protruding female gametophyte (Higashiyama et al., 2003; Higashiyama and Yang, 2017; Lopes et al., 2019).

Signals involved in pollen tube growth and guidance such as starch, AGPs, and GABA have been extensively studied in porogamous species. However, signaling molecules controlling the pollen tube journey have been overlooked in species in which the pollen tube entry into the ovule takes place through the chalaza, located at the opposite side of the micropyle or other parts of the ovule. Since chalazogamy was first described in *Casuarina* by Treub in 1891 [cited in Maheshwari (1950)], it has been observed in several phylogenetically diverse species. Chalazogamy is frequent in the Fagales and Sapindales. Examples in the Fagales include *Juglans regia* (walnut) (Nawaschin, 1895; Langdon, 1934; Nast, 1935, 1941; Luza and Polito, 1991), *Carya* (Billings, 1903; Langdon, 1934), *Ticodendron* (Sogo and Tobe, 2008), *Casuarina* (Sogo et al., 2004b), *Alnus* (Sogo and Tobe, 2005), or *Gymnostoma* (Sogo et al., 2004a). Examples in the Sapindales include the genus *Pistacia* (Copeland, 1955; Bradley and Crane, 1975; Grundwag, 1976; Shuraki and Sedgley, 1997; Martinez-Palle and Herrero, 1998) and *Mangifera indica* (mango) (de Wet et al., 1986). In the case of walnut, early pollination results in the presence of an underdeveloped ovule upon pollen tube arrival, and the pollen tubes are unable to enter into the micropyle, resulting in chalazogamy (Luza and Polito, 1991). In the case of pistachio (*Pistacia vera*), a ponticulus develops bridging the growth of the pollen tube from the style to the ovule (Martinez-Palle and Herrero, 1995). However, the ovary guidance signals involved in this alternative pollen tube pathway that eventually will cause its growth through the chalaza or other parts of the ovule, but not the micropyle, are unknown.

Mango is one of the most important fruit crops, ranking fifth worldwide in production after bananas, apples, grapes, and citrus (FAO, 2018). Mango is an andromonoecious evergreen tropical tree with inflorescences that are branched terminal panicles (Ramírez and Davenport, 2010) with a high number of flowers with a variable proportion of hermaphrodite and male flowers. Mango flowers are small, ranging in size from 5 to 10 mm in diameter (Ramírez and Davenport, 2016). The ovary has one chamber that contains a single anatropous ovule (Robbertse et al., 1986), and the flowers show chalazogamy (Joel and Eisenstein, 1980; de Wet et al., 1986). Because of its agronomic importance and its low fruit set (Singh, 1960), most of the studies on reproductive biology have been focused on optimizing tree management and production, especially on the effect of the environment on pollen germination (de Wet et al., 1989; Dag et al., 2000; Ramírez and Davenport, 2010; Pérez et al., 2019). Indeed, very few studies have focused on the essential process of the pollen tube pathway in this species, which is a key aspect for fertilization and, consequently, fruit production, further taking into consideration its partially unexplored unusual pollen tube growth. To fill this gap and with the goal of elucidating the support provided by the pistil to the unusual pollen tube growth in chalazogamous species, we perform a detailed study on the pollen tube pathway in mango, paying special attention to three molecules shown to play a key role in pollen tube growth of porogamous species. First, we study starch, which has a key nutritive role for pollen tube growth and can be considered as a marker of the pollen tube pathway in the style. Second, we evaluate JIM8 and JIM13 signals that recognize carbohydrate epitopes present in some specific AGPs, which have a nutritive and guiding role in pollen tube growth. Although different AGP epitopes can be recognized by using a range of monoclonal antibodies, in most of the porogamous species studied, the presence of relevant AGPs involved in pollen–pistil interaction has been revealed by using JIM8 and JIM13 monoclonal antibodies (Coimbra and Duarte, 2003; Losada and Herrero, 2012, 2014, 2017; Losada et al., 2014, 2017; Leszczuk et al., 2019). Finally, we analyze GABA, one of the few known molecules involved in pollen tube guidance observed in the micropyle of porogamous species, but not yet analyzed in chalazogamous species.

## Materials and Methods

### Plant Material and Pollinations

Flowers were collected from adult mango trees (*M. indica* L.) of the cultivars “Kent” and “Keitt” located in a mango collection at the IHSM-La Mayora-CSIC-UMA (Málaga, Spain), at latitude 36°45′N, longitude 4°4′W, and altitude 35 m above sea level. Hermaphrodite and male flowers were collected in the morning, from 08:30 to 10:30 h, before anther dehiscence. Then, these flowers were placed on wet white paper inside a Petri dish in the laboratory, where anther dehiscence occurred between 11:00 and 15:00 h. These flowers were used in the pollination experiments in which one hermaphrodite flower, which had been previously emasculated by removing the stamens, was pollinated by touching the stigma with the open anther of a single male or hermaphrodite flower (Pérez et al., 2016). For field experiments, hand pollination was performed on the trees. For laboratory experiments, the hand-pollinated hermaphrodite flowers were maintained on wet white paper inside Petri dish at room temperature.

### Microscopic Preparations

To observe the gynoecium anatomy, pistils from mature hermaphrodite flowers were fixed in 2.5% glutaraldehyde in 0.03 M phosphate buffer (Sabatini et al., 1963), dehydrated in a graded ethanol series, and embedded in Technovit 7100 resin (Heraeus Kulzer, Wehrheim, Germany). Embedded material was sectioned at 2 μm and stained with acridine orange 0.01% in water (Nicholas et al., 1986). Pistil anatomy was also observed in preparations stained with acridine orange 0.01% in water (Nicholas et al., 1986), from flowers previously fixed in formalin–acetic acid–alcohol (FAA).

To evaluate pollen tube growth and kinetics, hand-pollinated hermaphrodite flowers were fixed in FAA at 4, 8, 12, 16, 20, and 24 h after pollination. The pistils were washed three times for 1 h with distilled water and left in 5% sodium sulfite at 4°C for 24 h. Then, to soften the tissues, they were autoclaved at 1 kg/cm^2^ during 5 min in sodium sulfite (Jefferies and Belcher, 1974). To visualize pollen tubes, pistils were squashed and stained with 0.005% aniline blue in 0.15 N PO_4_K_3_ (O’Brien and McCully, 1981). The number of pollen tubes along the stigma, style, and ovule was counted from 26 pistils of the cultivar “Keitt.” Pollen tubes were also observed in resin embedded material that was sectioned at 2 μm and stained with periodic acid–Schiff reagent (PAS) (Feder and O’Brien, 1968).

To examine integument development, flower buds were collected at a range of different developmental stages, from bud differentiation up to mature flower. The flower buds were fixed in FAA for squash preparation and in 2.5% glutaraldehyde in 0.03 M phosphate buffer for resin sections (Sabatini et al., 1963). The squash preparations were stained with acridine orange 0.01% in water (Nicholas et al., 1986) and resin sections with PAS (Feder and O’Brien, 1968).

To observe starch grains in semithin sections from pollinated and unpollinated flowers, 2-μm resin sections were stained with PAS (Feder and O’Brien, 1968). Data were collected from 17 flowers in anthesis before pollination, 27 pollinated flowers 1 day after anthesis, and 13 unpollinated flowers 1 day after anthesis.

To observe vascular bundles, 2-μm resin sections were also stained for cutin with 0.01% auramine in 0.05 M phosphate buffer (Feder and O’Brien, 1968).

Preparations were observed under an epifluorescence Leica DM LB2 microscope with 340- to 380-nm bandpass and 425-nm long-pass filters for aniline blue, acridine orange, and auramine.

### Immunocytochemistry

For immunocytochemistry, ovules of mango from pollinated and unpollinated flowers were fixed in 4% paraformaldehyde in phosphate-buffered saline (PBS) at pH 7.3, left overnight at 4°C, dehydrated in an acetone series, embedded in Technovit 8100 (Kulzer), polymerized at 4°C, and sectioned at 2 μm. Sections were placed in a drop of water on a slide covered with 2% 3-aminopropyltrietoxysilane and dried at room temperature (Satpute et al., 2005; Solís et al., 2008). Different antibodies were used to localize specific cell components. Thus, JIM8 (Pennell et al., 1991) and JIM13 (Knox et al., 1991) rat monoclonal antibodies (Carbosource Service, University of Georgia, United States), were used to recognize AGPs, and an anti-GABA rabbit polyclonal antibody (Sigma, A2052) was used for GABA. Following the protocol of Lora et al. (2009), sections were incubated with PBS for 5 min and later with 5% bovine serum albumin in PBS for 5 min. Then, different sections were incubated for 1 h with the primary antibodies: JIM8 and JIM13 undiluted and anti-GABA diluted 1/25 in PBS. After three washes in PBS, the sections were incubated for 45 min in the dark with the corresponding secondary antibodies (anti-rat, for JIM8 and JIM13 and anti-rabbit, for anti-GABA) conjugated with Alexa 488 fluorochrome (Molecular Probes, Eugene, OR, United States) and diluted 1/25 in PBS. After three washes in PBS, the sections were stained with 4,6-diamidino-2-phenylindole (DAPI) (0.1 mg/mL) and washed three times in PBS. The sections were mounted in ProLong Gold Antifade Reagent (Invitrogen) and examined with a Leica DM LB2 epifluorescence microscope connected to a Leica DFC310 FX camera. Filters were 470/525 nm for the Alexa 488 fluorescein label of the antibodies, and a 340–380-nm excitation filter and an LP425 barrier filter for DAPI. Overlapping photographs were obtained with the Leica Acquisition Station AF6000 E.

### *In vitro* Pollen Tube Growth

To evaluate the effect of GABA on pollen germination and pollen tube elongation, we followed an *in vitro* protocol previously developed for mango that used a medium with 23 PEG 8000, 10% sucrose, 0.2 g/L MgSO_4_7H_2_O, 0.1 g/L KNO_3,_ 0.1 g/L H_3_BO_3_, and 0.7 g/L Ca(NO_3_)_2_4H_2_O (Pérez et al., 2019). To this germination medium, different concentrations ranging from 1 μM to 500 mM of GABA were tested. Pollen was considered as germinated when the length of the tube was longer than the grain diameter. Pollen tube length was quantified using the Fiji program (Schindelin et al., 2012), which is a version of ImageJ. Data were collected from three Petri dishes with at least 200 pollen grains or 100 pollen grains for pollen germination or pollen tube length, respectively, in each of four biological independent replicates.

### Statistical Analysis

The immunofluorescence signal was measured as relative fluorescence units (RFUs) using the Fiji program (Schindelin et al., 2012), and significant differences were evaluated using the Student *t* test, *P* < 0.05. RFUs were collected from at least three biological independent replicates. The statistical analysis of pollen germination and pollen tube elongation was performed with an analysis of variance, and Duncan multiple-range test was used to separate means (*P* ≤ 0.05). The percentages of pollen germination were arcsine–square root transformed for the Duncan multiple-range test. Statistical analyses were performed with the SPSS 15.0 statistical software.

## Results

### Pistil Anatomy and Pollen Tube Growth

**FIGURE 1:**
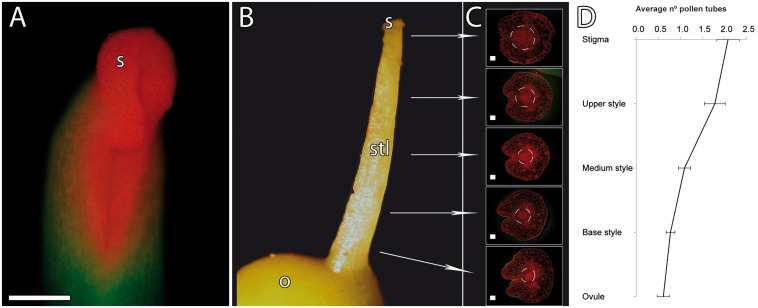
Pistil anatomy and pollen tube numbers along the pistil in mango. **(A)** Stigma and style stained with acridine orange. The stigma shows a horseshoe-shaped morphology stained in orange. **(B)** Pistil anatomy. **(C)** Gradual reduction of the transmitting tissue of the style (as shown with acridine orange staining of 2-μm transverse resin sections). **(D)** Gradual reduction of the number of pollen tubes along the stigma, style, and ovule (*n* = 99). Error bars indicate standard errors. Data were collected from 24 pistils. S, stigma; STL, style; O, ovary. **(A)** Scale bar, 100 μm. **(C)** Scale bars, 20 μm.

To define the unusual pathway of the pollen tubes in mango, we first evaluated the pistil anatomy of the hermaphrodite flower, with special attention to the transmitting tissue, and pollen tube growth. The mango pistil consists of a single carpel, showing a recognizable suture line that extends from the horseshoe-shaped stigma to the base of the style (Bachelier and Endress, 2009; Figures 1A,B). A transmitting tissue runs inside the pistil, and a progressive reduction of the diameter of the transmitting tissue toward the base of style was observed (Figure 1C), resulting in the reduction of the number of pollen tubes along the styles (Figure 1D).

**FIGURE 2:**
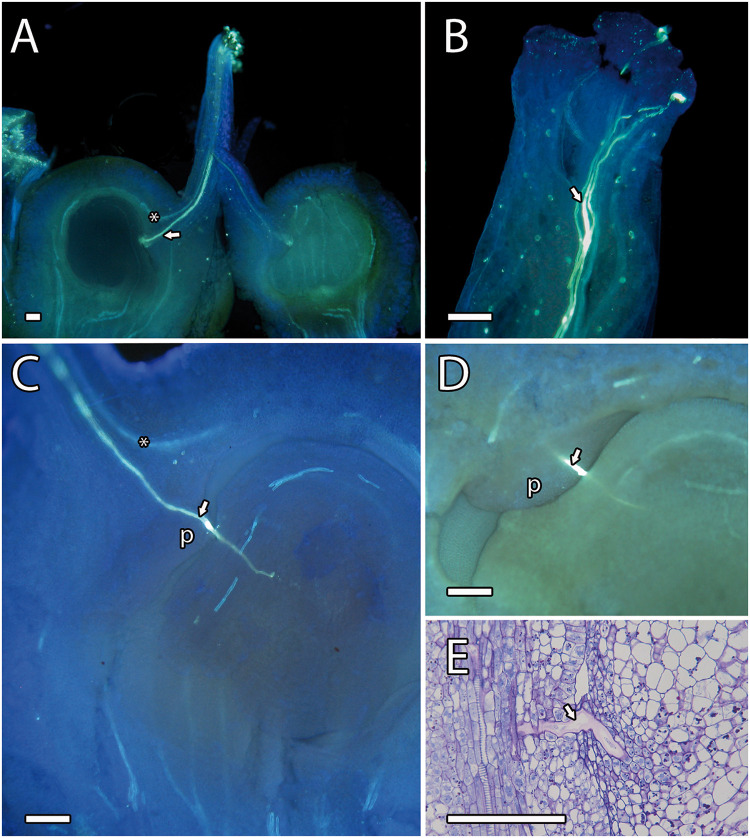
Pollen tube growth in mango. **(A)** Pistil showing a pollen tube (arrowhead) through the style close to the vascular bundles (asterisk) and the ponticulus. **(B)** Pollen tubes (arrowhead) growing on the stigma. **(C)** Pollen tube growing along the ponticulus, the distal part of funiculus, and reaching the nucellus. The asterisk indicates the vascular bundles. **(D,E)** Close-up of a pollen tube (arrowhead) growing along the ponticulus detected by aniline blue **(D)** and PAS and toluidine blue **(E)** staining. **(A–D)** Aniline blue staining of squash preparations. P, ponticulus. Scale bars, 100 μm.

**FIGURE 3:**
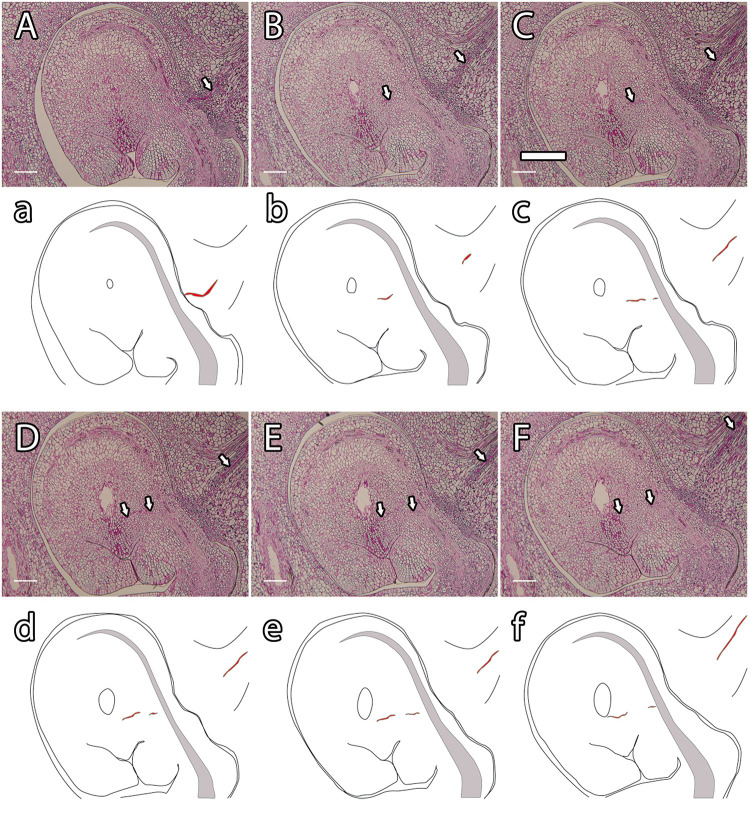
Histological observation of pollen tube growth in mango **(A–F)** and its schematic representation **(a–f)**. A pollen tube (arrowhead and red line) growing along the stylar transmitting tissue **(A; a)**, the ponticulus, the distal part of the funiculus **(B,C; b,c)**, the nucellus **(D–F; d–f)** and reaching the embryo sac **(F; f)**. **(A–F)** PAS staining. Scale bars, 100 μm.

After pollination, a fast hydration and germination of the pollen grains on the stigma were observed. Pollen tubes were observed in the upper part of the style 4 h after pollination, reaching the ovule 24 h after pollination. We observed pollen tubes growing along the stylar transmitting tissue close to the vascular bundles (Figures 2A,B). At the base of the style, while the vascular bundles surrounded the ovule, the pollen tubes went straight to the ponticulus, a protuberance of the ovary that acts as a bridge between the tissues of the ovary and ovule (Figures 2C–E) [also reported in pistachio by Martinez-Palle and Herrero (1995)]. Pollen tubes continued growing in the ovule through the distal part of the funiculus and the proximal end of the integument and the raphe chalaza as previously reported by Robbertse et al. (1986). Pollen tubes reached the lateral side of the nucellus and grew through the nucellus to reach the embryo sac (Figure 3).

**FIGURE 4:**
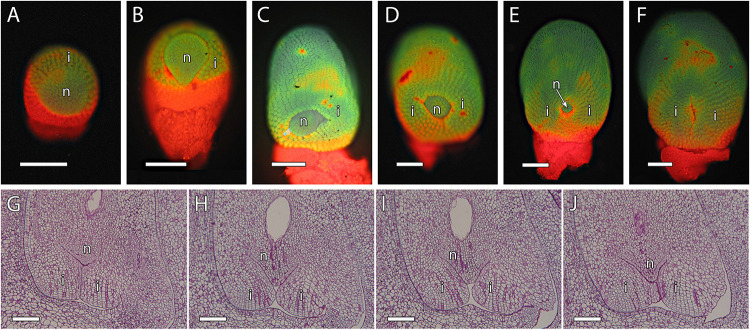
Integument development in mango ovules. **(A)** Early stage of ovule development showing the emerging integument. **(B–F)** At later stages, the nucellus is gradually surrounded by the integument. **(G–J)** Serial 2-μm resin sections of the micropyle at anthesis. **(A–F)** Acridine orange staining of squash preparation. **(G–J)** PAS staining. N, nucellus; I, integument. Scale bars, 100 μm.

Fertilization requires synchronization of the male gametophyte with the receptivity stage of ovule development. As chalazogamy has been related to a not fully developed ovule (Luza and Polito, 1991), we first evaluated ovule development in mango. The integument was almost imperceptible at early stages of ovule development (Figure 4A). Later on, we observed only a single integument starting to partially wrap around the nucellus that was nearly complete at anthesis (Figures 4B–F). Because in flowering plants pollen tube entry into the ovule generally takes place through the micropyle (Lora et al., 2019), we specifically studied the developmental stage of the micropyle by analyzing serial semithin sections of the ovule. The semithin sections confirmed a single integument in mango. Although several semithin sections showed a closed micropyle, a detailed study of serial sections of ovules revealed an open micropyle at anthesis that was also observed 1 day after anthesis (Figures 4, 7A,C), suggesting that the ovules were not fully developed at anthesis.

### Changes in Starch and AGPs in the Pistil

**FIGURE 5:**
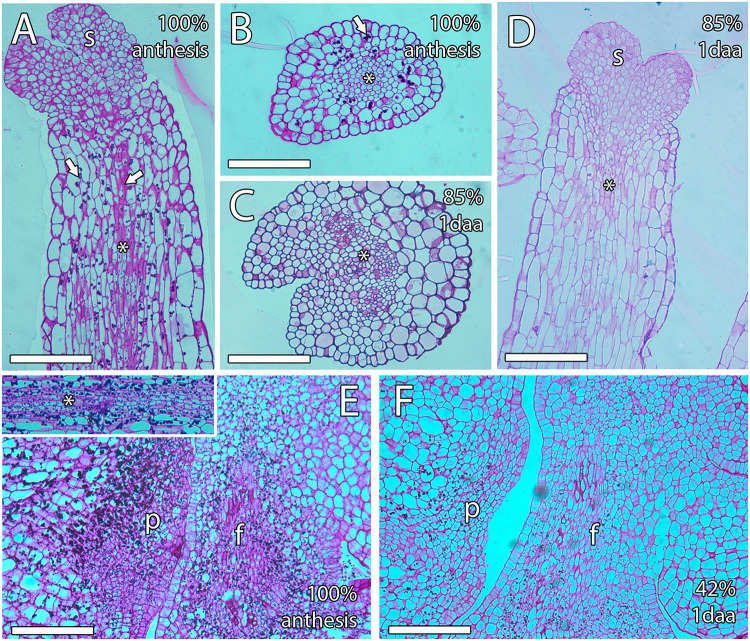
Starch grains along the transmitting tissue of mango pistils. **(A,B)** Longitudinal **(A)** and transversal **(B)** sections of the style at anthesis showing big starch grains (arrow) around the stylar transmitting tissue (asterisk), which contains smaller starch grains (arrow). **(C,D)** Transversal **(C)** and longitudinal **(D)** sections of the style 1 day after anthesis showing a small amount of starch grains. The asterisk indicates the stylar transmitting tissue. **(E)** The ponticulus is full of starch grains at anthesis. The upper square shows starch grains in the transmitting tissue (asterisk) in the proximity of the ponticulus at anthesis. **(F)** The ponticulus contains less starch grains 1 day after anthesis. S, stigma; P, ponticulus; F, funiculus. Scale bars, 100 μm.

To further understand the non-porogamous pollen tube growth in mango, we next studied several molecules that have been reported as key for pollen tube growth in porogamous species. Among them, starch grains have been proposed to support heterotrophic pollen tube growth in the pistil and, consequently, are generally found along the pollen tube pathway. We evaluated semithin sections of ovules stained with PAS reagent. At anthesis, PAS staining revealed dense PAS reagent–positive cytoplasm and small starch grains in the cells of the transmitting tissue of the style and bigger starch grains in the cells around the transmitting tissue (100% *n* = 15, Figures 5A,B). However, the number of starch grains and the PAS reagent–positive cytoplasm in the style decreased in the days following anthesis regardless of pollination. Thus, 1 day after anthesis, in pollinated and unpollinated flowers, 85% (*n* = 27) and 100% (*n* = 13), respectively, of the analyzed ovules showed almost no starch grains (Figures 5C,D). Similarly, starch grains and dense PAS reagent–positive cytoplasm were specifically observed in the cells of ponticulus of all the analyzed ovules (*n* = 17) from flowers at anthesis (Figure 5E) but were almost absent in 42% (*n* = 19) of the analyzed ovules from pollinated flowers 1 day after anthesis (Figure 5F).

**FIGURE 6:**
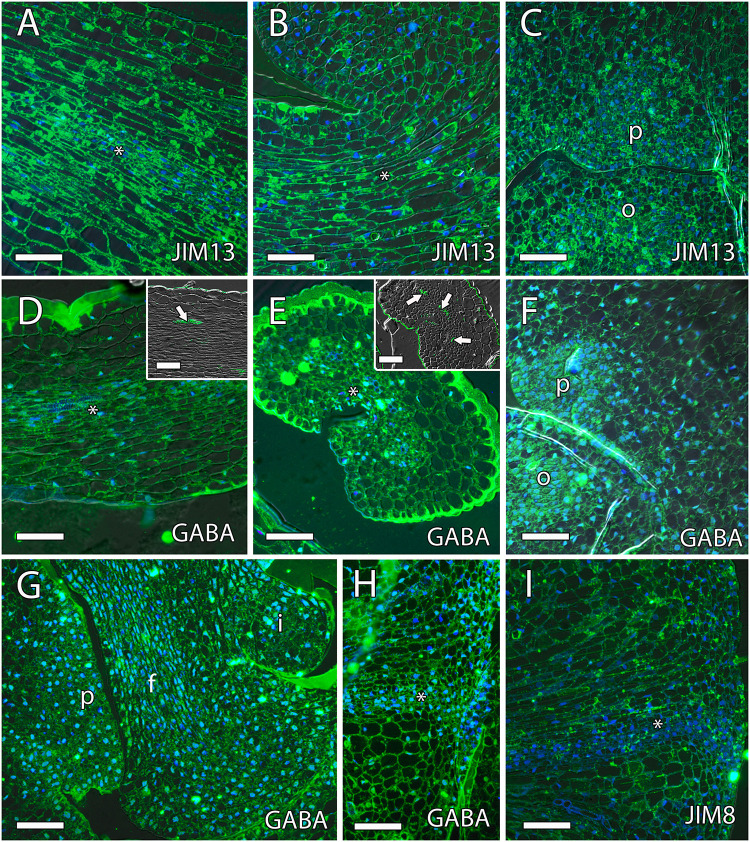
γ-Aminobutyric acid (GABA) and arabinogalactan proteins (AGPs) along the transmitting tissue in mango pistils. **(A–C)** JIM13-specific labeling (AGPs) were homogenously observed in the transmitting tissue (asterisk) in the style [middle part **(A)** and the base of the style **(B)**] and ponticulus **(C)** at anthesis. **(D–F)** Anti-GABA labeling (GABA) was observed differently in longitudinal **(D)** and transversal **(E)** sections of the transmitting tissue (asterisk) and in the ponticulus **(F)** at anthesis. **(D,E)** Vascular bundles in the style stained with auramine in the upper squares. **(G,H)** Anti-GABA labeling was also observed in the ponticulus 1 day after anthesis in pollinated **(G)** and unpollinated **(H)** flowers. The asterisk indicates the transmitting tissue. **(I)** JIM8-specific labeling (AGPs) were homogenously observed in the ponticulus 1 day after anthesis in unpollinated flowers. The asterisk indicates the transmitting tissue. **(A–I)** Immunolocalization of GABA and AGPs in the pistil of mango flowers were revealed by anti-GABA polyclonal antibody for GABA and JIM8 and JIM13 monoclonal antibodies for AGPs. P, ponticulus; O, ovule; I, integument, F, funiculus. Scale bars, 50 μm.

**FIGURE 7:**
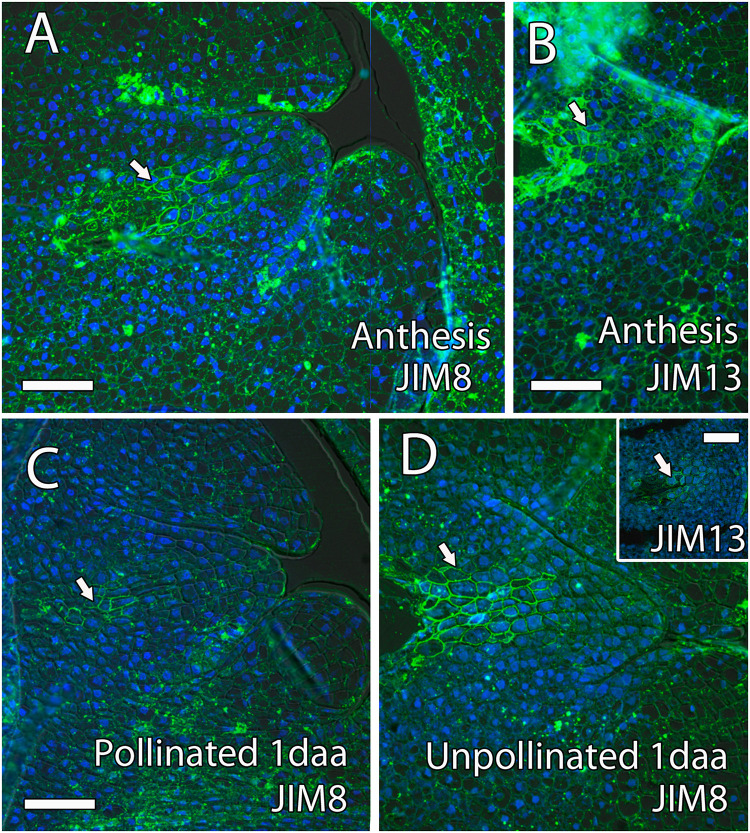
Arabinogalactan proteins (AGPs) in the distal part of the mango ovule. **(A)** JIM8- and **(B)** JIM13-specific labeling (AGPs) was observed in the distal part of the nucellus (arrow) around the embryo sac at anthesis. **(C,D)** Similarly, AGPs reacted against JIM8 antibody in the distal part of the nucellus (arrow) around the embryo sac in pollinated **(C)** and unpollinated **(D)** flowers 1 day-after-anthesis (daa). The upper square shows JIM8-specific labeling in the distal part of the nucellus (arrow) around the embryo sac in unpollinated flowers 1 day after anthesis. **(A–D)** Immunolocalization of AGPs in the ovule of mango flowers was revealed by JIM8 and JIM13 monoclonal antibodies. Scale bars, 50 μm.

Arabinogalactan proteins have also been previously reported as key molecules for pollen tube growth in the stigma, style, and micropyle in different species (Pereira et al., 2016). The AGPs that reacted positively to JIM8 and JIM13 were similar, without appreciable differences. Both antibodies were uniformly observed in the ovule, the style, and the ponticulus (Figures 6A–C). JIM8 and JIM13 signal that was measured as RFUs was similar in the transmitting tissue, including the ponticulus, and the surrounding cells (*t*_8_ = 1.42, *P* > 0.05 for JIM8, and *t*_4_ = 1.04, *P* > 0.05, for JIM13 in the ponticulus of flowers in anthesis; *t*_8_ = 0.42, *P* > 0.05, for JIM8 in the style of pollinated flowers 1 day after anthesis). AGPs that reacted to JIM8 and JIM13 were specifically detected in the distal part of the nucellus, next to the micropyle, and in direct contact with the embryo sac, in the ovules from flowers in anthesis (Figures 7A,B), 1 day after pollination (Figure 7C) and 1 day after anthesis in unpollinated flowers (Figure 7D).

### GABA Along the Pollen Tube Pathway

Recently, the role of GABA and the integuments on pollen tube growth has been reviewed (Lora et al., 2019). The results show that, while low levels of GABA stimulate *in vitro* pollen tube growth, high levels of GABA in the inner integument prevent the entrance of multiple pollen tubes into the ovule (Palanivelu et al., 2003; Lora et al., 2019). Immunolocalization of GABA using the polyclonal anti-GABA antibody showed that GABA specifically reacted with anti-GABA in the transmitting tissue of the style, in the ponticulus, and in the funiculus of flowers in anthesis and 1 day after anthesis in both pollinated and unpollinated flowers. The signal of anti-GABA was measured as RFUs and revealed significant differences between the ponticulus and the surrounding cells (*t*_12_ = 5.8, *P* < 0.05, and *t*_8_ = 4.5, *P* < 0.05, for anti-GABA signal in the ponticulus of flowers in anthesis and 1 day after anthesis, respectively). The analysis of variance of RFUs between the transmitting tissue and the surrounding cells in the style was slightly higher (*P* = 0.09) of the limit of significance in anthesis (*t*_8_ = 1.9, *P* > 0.05) but was significantly different 1 day after anthesis in unpollinated flowers (*t*_4_ = 3.09, *P* < 0.05). However, high levels of anti-GABA signal were not observed in the single integument of the ovule (Figure 6 and Supplementary Figure 1).

**FIGURE 8:**
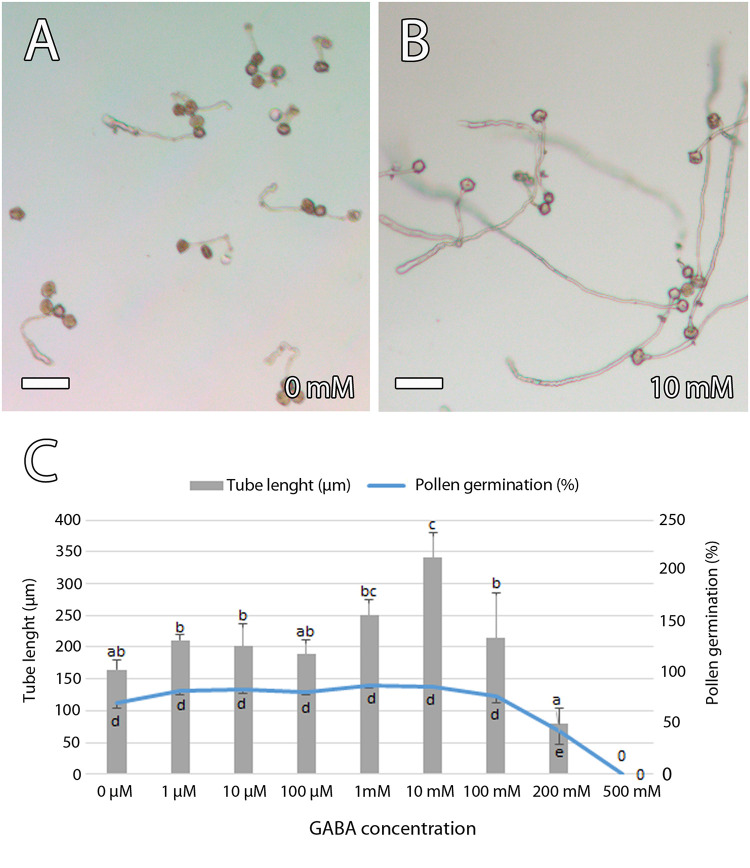
Effect of γ-aminobutyric acid (GABA) on *in vitro* mango pollen germination. **(A,B)** Short pollen tube in a germination medium without GABA **(A)** compared to the long pollen tubes that were observed in a germination medium containing a GABA concentration of 10 mM **(B)**. **(C)** GABA stimulated *in vitro* pollen tube length that was significantly higher in a germination medium containing a GABA concentration of 10 mM. Error bars indicate standard errors. Data were collected from four biological independent replicates. Means followed by different letters are significantly different (*p* ≤ 0.05) by Duncan multiple-range test. Scale bars, 100 μm.

To further evaluate the role of GABA on pollen tube growth, we analyzed the effect of different concentrations of GABA on pollen tube growth and elongation *in vitro*. To simulate the possible GABA concentrations in the mango carpel, we included the range of GABA concentrations observed in the carpel of *Arabidopsis* wild type and *pop2* that range from 0.07 μM to 24 mM (Palanivelu et al., 2003). Pollen germination was 70% ± 6.1% (SE) and pollen tube elongation was 164 ± 15 μm (SE) in a germination medium without GABA. Differences in pollen germination among germination media containing different concentrations of GABA were significant only in the medium containing a GABA concentration of 200 mM, suggesting no effect of GABA on pollen germination. However, significant differences were observed for pollen tube elongation, reaching the greatest difference at a GABA concentration of 10 mM showing pollen tube elongation of 342 ± 37 μm (SE) (Figure 8). Taken together, the results of immunolocalization of GABA and *in vitro* pollen germination suggest a role for GABA in pollen tube growth and guidance in mango.

## Discussion

The kinetics of the pollen tube pathway in mango shows a conserved and rapid pollen germination and tube growth in the style (Pérez et al., 2016; Ramírez and Davenport, 2016). At the base of the style, the pollen tube reaches the ponticulus that shows starch grains and GABA. It enters laterally into the funiculus of the ovule and grows through the nucellus, reaching the egg apparatus. Mango, therefore, is chalazogamous and shows an unusual pollen tube growth in angiosperms in which most of the species show porogamy.

### Chalazogamy From an Evolutionary and Developmental Perspective

When chalazogamy was first reported by Treub in 1891 (Maheshwari, 1950), it was just considered as a valuable feature for taxonomic classification. However, additional observations showed the great diversity in the mode of pollen tube entry into the ovule in chalazogamous species, including mango in which pollen tubes grow through the distal part of funiculus and the proximal end of the integument and the raphe chalaza (Robbertse et al., 1986). Therefore, chalazogamy was increasingly considered as a physiological feature (Maheshwari, 1950). Chalazogamy has been mainly observed in the Fagales (Sogo and Tobe, 2008) and Sapindales (Martinez-Palle and Herrero, 1998). The study on pollen tube growth in several species in the Fagales revealed the apomorphic feature of chalazogamy in this order, whereas porogamy seems to be plesiomorphic (Sogo and Tobe, 2008). Because chalazogamy has been found in unrelated species in Sapindales such as pistachio (Martinez-Palle and Herrero, 1998) or mango, it is expected that chalazogamy is also apomorphic in the Sapindales and in angiosperms as a whole.

The presence of chalazogamy has been associated with the underdeveloped stage of the ovule (Luza and Polito, 1991; Martinez-Palle and Herrero, 1995; Sogo et al., 2004b). A similar observation was made in our work as hermaphrodite flowers showed ovules with non–fully developed micropyles at anthesis. It is also expected that the ovary signal for pollen tube growth and guidance should be synchronized with the pollen tube arrival (Herrero, 2003). However, the knowledge is scarce on ovary signals for the unusual pollen tube growth in chalazogamous species.

### A Conserved Pollen Tube Pathway in the Style

The first meeting place between the male gametophyte and the female sporophyte occurs in the stigma. Later on, the style provides an adequate environment for pollen tube competition in compatible crosses that is even more evident in species showing a long style (Hormaza and Herrero, 1992; Herrero and Hormaza, 1996; Mazer et al., 2016). Thus, pollen tube competition in the style is favored by the physical constraint caused by the reduction along the style of the diameter of the transmitting tissue. This is a conserved feature in angiosperms (Herrero and Hormaza, 1996; Hormaza and Herrero, 1996; Lora et al., 2016) that was also observed in mango. As a consequence, there is a gradual reduction in the number of pollen tubes along the style, such as has been reported in *Primula obconica* (Modlibowska, 1942), *Nicotiana glauca* (Cruzan, 1986), *Prunus avium* (Hormaza and Herrero, 1996), *Polygala varyedae* (Castro et al., 2009), or *Andansonia* species (Rasoamanana et al., 2019).

In addition to this physical constraint, the pollen tubes also compete for the stylar reserves that allow the heterotrophic growth of the pollen tubes (Herrero and Dickinson, 1979; Hormaza and Herrero, 1996; Gotelli et al., 2017). Indeed, starch grains are generally observed in the transmitting tissue of porogamous species (Herrero and Dickinson, 1979; Herrero and Hormaza, 1996; Lora et al., 2016). Similarly, ultrathin sections of the style of mango revealed a stylar exudate that stained for polysaccharides and lipids (de Wet et al., 1990). We observed starch grains in the style and the ponticulus that define the unusual pollen tube pathway of mango. The nutritive role of those starch grains is suggested, not only because they are observed along the pollen tube pathway, but also because in different species such as the kiwifruit (Gonzalez et al., 1996) those starch grains decrease after the passing of the pollen tube. Interestingly, the number of starch grains also decreased 1 day after anthesis in mango, supporting the role of the starch grains on the heterotrophic growth of pollen tubes in this species.

The style supports pollen tube growth but has also a guidance effect for pollen tubes using several molecules (Lamport et al., 2018). Among them, AGPs have been reported as a key player for pollen–pistil interaction in several porogamous species (Coimbra and Duarte, 2003; Losada and Herrero, 2012; Losada et al., 2014, 2017; Leszczuk et al., 2019). AGPs are extensively glycosylated Hyp-rich proteins, and Wu et al. (1995) reported a glycosylation gradient from the stigma to the base of the style in *Nicotiana tabacum*. Moreover, AGPs stimulate *in vitro* pollen tube growth in several species (Cheung et al., 1995; Mollet et al., 2002; Losada et al., 2017). We observed AGPs detected by JIM8 and JIM13 along the whole style of mango, but there was not a specific accumulation in the transmitting tissue in contrast with the immunolocalization of GABA that was present along the transmitting tissue of the style. GABA has a clear effect on pollen tube growth *in vitro*: high GABA levels inhibit pollen tube growth, whereas low GABA levels stimulate it. Because high GABA levels have been observed in the inner integument that forms the micropyle of porogamous *Arabidopsis* (Palanivelu et al., 2003) and *Annona cherimola* (Lora et al., 2019), an inhibitory role on pollen tube growth and prevention of multiple pollen tube entry into the ovule has been suggested. In the case of mango, pollen tube entry into the ovule does not take place through the micropyle, but through the ponticulus, which is where differences appear between porogamous and chalazogamous species.

### Differences Begin at the Ovary Locule

Once pollen tubes have reached the base of the style, they grow through the ponticulus, a protuberance of the ovary wall that bridges the ovary and the ovule, as described by de Wet et al. (1986) and Joel and Eisenstein (1980). This structure is common to other species of the Anacardiaceae (Bachelier and Endress, 2009), including pistachio (*P. vera*), in which the ponticulus has been shown as a funicular protuberance in close proximity to the stylar base (Martinez-Palle and Herrero, 1995, 1998). It has been suggested that the formation of the ponticulus of mango is associated with pollination (Joel and Eisenstein, 1980), but we have observed the ponticulus in both pollinated and unpollinated flowers, indicating that ponticulus formation in mango is a developmentally regulated process independent on pollination. A similar situation has been observed in pistachio (Martinez-Palle and Herrero, 1995).

A similar protuberance of the ovary wall, the obturator, involved in the pollen tube access to the micropyle, has been reported in some porogamous species such as peach (*Prunus persica*) (Arbeloa and Herrero, 1987), kiwifruit (*Actinidia deliciosa*) (Gonzalez et al., 1996), or apple (*Malus* × *domestica*) (Losada and Herrero, 2017). Starch grains have been observed in the obturator of these species. Likewise, starch grains were observed in the ponticulus of mango. The similarity between the obturator and the ponticulus suggests a similar function of the ponticulus in regulating pollen tube access to the ovule.

Interestingly, similar to the observation in the stylar transmitting tissue, we also observed GABA detected by anti-GABA in the ponticulus. Moreover, GABA stimulates *in vitro* pollen tube elongation in mango, as has been reported in others species (Palanivelu et al., 2003; Ling et al., 2013; Yu et al., 2014; Lora et al., 2019). Thus, our results suggest that GABA could play a role in signaling for the growth and guidance of the pollen tube in mango toward the ovule. Indeed, a few years ago, a mechanism by which GABA affects pollen tube growth *via* modulating Ca^2+^-permeable membrane channels was reported in *Nicotiana* (Yu et al., 2014). However, while GABA was specifically observed in the inner integument of porogamous *A. cherimola* (Lora et al., 2019) and *Arabidopsis* (Palanivelu et al., 2003), it has not been observed in the single integument of chalazogamous mango. This observation could reflect the loss of the function of the micropyle in the support and guidance of the pollen tubes in mango, and this could be related to the unitegmy of this species.

Besides the signal from the micropyle in porogamous species, the signals from the embryo sac are key for pollen tube growth and guidance (Higashiyama et al., 2003; Higashiyama and Yang, 2017; Lopes et al., 2019). To our knowledge, there are no studies on ovular guidance in chalazogamous species, but because pollen tube entry into the embryo sac always takes place through the distal part, we would also expect a key role of the embryo sac on pollen tube guidance and growth in mango. Indeed, we observed pollen tube growing into the distal part of the nucellus and reaching the embryo sac. In these cell layers of the nucellus, we observed AGPs. A similar AGP distribution was observed in the crassinucellate ovules of porogamous species such as *A. cherimola* (Lora et al., 2019), *Malus domestica* (Losada and Herrero, 2019), and *Amaranthus hypochondriacus* (Coimbra and Salema, 1997). In the tenuinucellate ovules of *Arabidopsis*, where the mature embryo sac shows a single nucellus cell layer between the megasporophyte and the epidermal cells, AGPs detected by JIM8 and JIM13 were observed in the tip of the inner integument and embryo sac (Coimbra et al., 2007). Similarly to the situation in the stigma and the style, the AGPs around the distal part of the embryo sac could have a similar role for pollen tube growth and guidance, although in this case, no differences would be expected between porogamous and chalazogamous species.

In summary, in contrast to the observation in porogamous species, immunolocalization did not reveal AGPs and GABA in the micropyle of the mango ovule, (Lora et al., 2019). AGPs, detected by JIM8 and JIM13 antibodies, were observed in the distal part of the nucellus around the embryo sac and GABA along the transmitting tissue, including the ponticulus. Thus, ovary signals could be adapted to the pollen tube–ovule interaction in mango. It will be worth to evaluate if the ovary signals here observed in mango are also conserved in other chalazogamous species and represent a more widespread situation in chalazogamous species.

## Data Availability Statement

The original contributions presented in the study are included in the article/Supplementary Material, further inquiries can be directed to the corresponding author/s.

## Author Contributions

JL, VP, MH, and JH conceived the study. JL, VP, MH, and JH designed the experiments and wrote the manuscript. JL and VP performed the experiments. All authors contributed to the article and approved the submitted version.

## Conflict of Interest

The authors declare that the research was conducted in the absence of any commercial or financial relationships that could be construed as a potential conflict of interest.

## Supplementary Material

Supplementary Figure 1 γ-Aminobutyric acid (GABA) and arabinogalactan proteins (AGPs) along the transmitting tissue in mango pistils. (A,B) Anti-GABA labeling (GABA) was observed differently in the ponticulus at anthesis. Immunolocalization of GABA was revealed by anti-GABA polyclonal antibody. P, ponticulus; O, ovule. Scale bars, 50 μm.
