# Supplementary figures and images for: Ovary Signals for Pollen Tube Guidance in Chalazogamous Mangifera indica L
Source: Front Plant Sci. 2021 Feb 10;11:601706. doi: 10.3389/fpls.2020.601706 (PMC7902493; doi:10.3389/fpls.2020.601706)

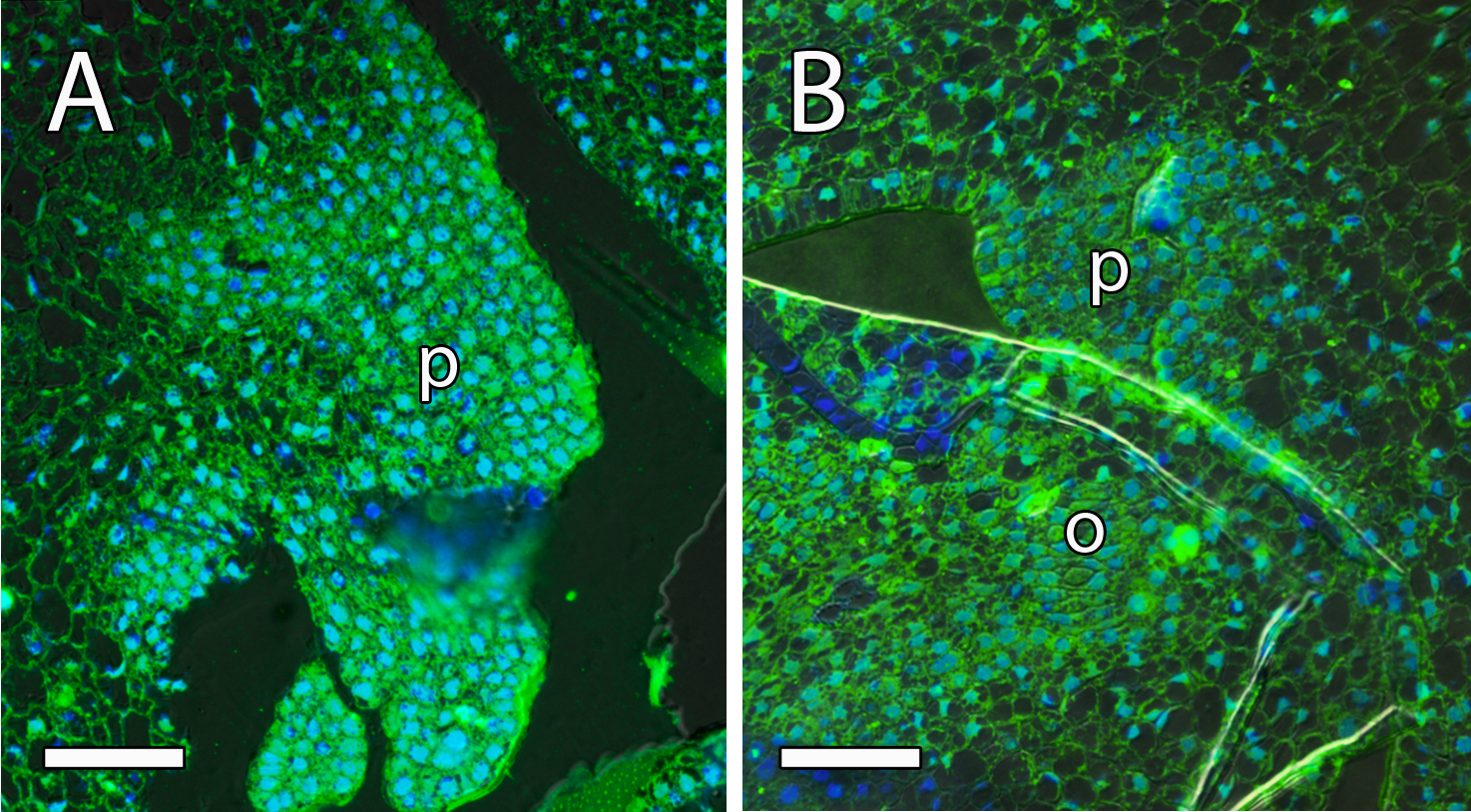

Supplement: Supplementary Figure 1 — γ-Aminobutyric acid (GABA) and arabinogalactan proteins (AGPs) along the transmitting tissue in mango pistils. (A,B) Anti-GABA labeling (GABA) was observed differently in the ponticulus at anthesis. Immunolocalization of GABA was revealed by anti-GABA polyclonal antibody. P, ponticulus; O, ovule. Scale bars, 50 μm. [file Image_1.tif]
